# Annotation of SBML models through rule-based semantic integration

**DOI:** 10.1186/2041-1480-1-S1-S3

**Published:** 2010-06-22

**Authors:** Allyson L Lister, Phillip Lord, Matthew Pocock, Anil Wipat

**Affiliations:** 1 Centre for Integrated Systems Biology of Ageing and Nutrition, Institute for Ageing and Health, Newcastle University, Campus for Ageing and Vitality, Newcastle upon Tyne NE4 5PL, UK; 2 School of Computing Science, Newcastle University, NE1 7RU, UK

## Abstract

**Background:**

The creation of accurate quantitative Systems Biology Markup Language (SBML) models is a time-intensive, manual process often complicated by the many data sources and formats required to annotate even a small and well-scoped model. Ideally, the retrieval and integration of biological knowledge for model annotation should be performed quickly, precisely, and with a minimum of manual effort.

**Results:**

Here we present rule-based mediation, a method of semantic data integration applied to systems biology model annotation. The heterogeneous data sources are first syntactically converted into ontologies, which are then aligned to a small domain ontology by applying a rule base. We demonstrate proof-of-principle of this application of rule-based mediation using off-the-shelf semantic web technology through two use cases for SBML model annotation. Existing tools and technology provide a framework around which the system is built, reducing development time and increasing usability.

**Conclusions:**

Integrating resources in this way accommodates multiple formats with different semantics, and provides richly-modelled biological knowledge suitable for annotation of SBML models. This initial work establishes the feasibility of rule-based mediation as part of an automated SBML model annotation system.

**Availability:**

Detailed information on the project files as well as further information on and comparisons with similar projects is available from the project page at http://cisban-silico.cs.ncl.ac.uk/RBM/.

## Background

### Annotation of systems biology models

A quantitative model of molecular systems describes the dynamics of the interactions between biological entities involved. Such modelling is central to systems biology. Models and experiments are typically refined iteratively: models provide useful feedback to experimentalists, who in turn pass their results back to improve the model. The creation of systems biology models, such as those written in Systems Biology Markup Language (SBML) [1] or CellML [2], is a primarily manual process. Making use of the many data sources and formats relevant to model development is time-consuming for modellers. While a small number of core databases can be used to retrieve a large amount of biological information relevant to the modeller, accessing the “long tail” of information stored in other resources is where manual processes become difficult.

Systems biology models can contain both the quantitative information required to run the simulation and biologically-meaningful annotation. However, while models often include the mathematical information required for simulations, they are not required to contain explicit information about the full biological context. Annotation provides a description of how a model has been generated and defines the biology of its components in a computationally-accessible fashion. While the presence of biological annotation aids efficient exchange, reuse, and integration of models, simulation does not require annotation and therefore such information is often limited or lacking. A well-annotated model is useful for simulation, as an input in other computational tasks and as a reference for researchers. A model with limited annotation is frequently useful only to the modeller who created it due to the often ambiguous naming schemes and lack of biological context [3,4].

Biological annotation in SBML is structured according to the Minimal Information Required in the Annotation of Biochemical Models (MIRIAM) specification [3]. There are three parts to MIRIAM: a recommended URI-based structure for compliant annotations, a set of resources to generate and interpret those URIs and a checklist of minimal information requested in the annotation of biological models. While other annotations are allowed within the specification, it is primarily the MIRIAM annotations which are relevant to the work presented here. MIRIAM annotations are URIs added to models in a standardised way, and which link external resources such as ontologies or data sources to a model. MIRIAM provides a standard structure for explicit links between the mathematical and biological aspects of a model. Aids to model annotation exist [5-9], but rely extensively on the expert knowledge of the modeller for identification of appropriate additions. There is a need for computational approaches that automate the integration of multiple sources to enable the model annotation process.

### Data integration in the life sciences

The integration of life sciences data remains an ongoing challenge due to the multitude of data sources and formats differing both in syntax and semantics. Errors in data integration can occur when data sources do not describe their information with a shared semantics [10]. The problems of, and historical approaches to, syntactic and semantic data integration have been well described [11,12]. Syntactic heterogeneity is when data of interest is available in different formats, and is generally resolved through the use of a common schema (such as with data warehousing techniques) or through translation of a single query into multiple queries understandable by each of the underlying data sources (query translation). Semantic heterogeneity describes the difference in the meaning of data among different data sources. A high level of semantic heterogeneity makes direct mapping difficult, often requiring further information to ensure a successful mapping. Semantic data integration is intended to resolve both syntactic and semantic heterogeneity and can allow a richer description of biology than is possible with syntactic methods, which often align data by linking structural units such as XSD components or table and row names.

An important concept in semantic data integration is* ontology -mapping*, which maps between classes in two or more ontologies [13]. In the simplest case of ontology mapping a source ontology is mapped directly onto a second, target, ontology. If only two ontologies need to be reconciled, ontology mapping is an effective strategy, as only two conceptualisations need to be compared. However, the complexity increases as more ontologies are added, rapidly limiting this method's usefulness. More complex mapping methods include *mediator*-based approaches, where a number of source ontologies are individually mapped to a single target, or *core ontology*. Usually, a core ontology is a description of the domain of interest rather than a description of the syntactic structure of the data. Previous work with ontology mapping and other semantic data integration methodologies in the life sciences [12,14-18] are discussed further at http://cisban-silico.cs.ncl.ac.uk/RBM/.

### Semantic data integration for model annotation

Systems biology model annotation can be time-consuming, as there are many resources for model annotation; the modeller may not even be aware of all of them. Accordingly, annotation is often slow and difficult. Current systems biology model annotation tools rely mainly on syntactic data integration methods such as query translation. While large amounts of data can be gathered and queried in this manner, the integration process is limited to resolving formatting differences without attempting to address semantic heterogeneity. While existing tools for model annotation are useful, semantic data integration methods can be used to resolve differences in the meaning of the data.

Therefore, in the work presented here we have developed rule-based mediation as an approach to semantic data integration for systems biology. In rule-based mediation, we use a set of* syntactic ontologies* which result from the conversion of a single data format into OWL constrained by Description Logics (OWL-DL) [19]. These syntactic ontologies are then individually linked to a core ontology, which describes the domain of interest. In rule-based mediation, a core ontology is completely independent of any of the syntactic ontologies, unlike traditional methods where one ontology type is created as a view of the other, such as pure global-as-view or local-as-view. However, rule-based mediation is similar to approaches such as the BYU Global-Local as View (BGLaV) [20]. Rule-based mediation allows both the straightforward addition of new syntactic ontologies as well as the maintenance of the core ontology as an independent entity.

In rule-based mediation, we* materialise* the data in the core ontology by using a set of rules to translate the data from the syntactic ontologies to the core ontology. This avoids the complex query translation algorithms required by the BGLaV approach.

In short, rule-based mediation has a number of defining features:

• the data from syntactically and semantically different data sources are materialised within a core ontology for easy access and stable querying;

• the core ontology is a semantically-meaningful model of the biological domain of interest;

• implementation can be performed using off-the-shelf tools and mapping languages.

## Results

### Rule-based mediation

In rule-based mediation, syntactic heterogeneity is resolved through the conversion of disparate data formats into syntactic ontologies. The data from the syntactic ontologies is then mapped to the core ontology, which stores the data in a semantically-homogeneous way. The core ontology, which describes the biological domain of interest, can then be queried and reasoned over. There are three main parts to rule-based mediation: the syntactic ontologies for describing the disparate data formats, the core ontology, and the mapping rules for linking the syntactic ontologies to the core ontology.

We have used standard languages for creating (e.g. Web Ontology Language (OWL) [19]), querying (e.g. Semantic Query-Enhanced Web Rule Language (SQWRL) [21]), and mapping (e.g. Semantic Web Rule Language (SWRL) [22]) ontologies. While other ontology languages such as the Open Biomedical Ontologies (OBO) [23] are also widely used in the life sciences, they do not provide the same level of support for automated semantic reasoning as OWL-DL [24].

#### Syntactic ontologies

As with other mediator-based approaches, in rule-based mediation the data formats are converted to syntactic ontologies. In our approach, the purpose of syntactic ontologies is to describe the data formats as well as isolate syntactic variability so that the core ontology models only the semantics of the data being integrated. Though this method can use any ontological format, here a syntactic ontology is a conversion of a non-OWL format such as XML into OWL-DL. Information from one or more data sources sharing the same data format is loaded into that format's syntactic ontology. A syntactic ontology used in this way allows both the import of data to the core ontology and the export of new information to the underlying data sources.

#### Core ontology

Often, mediator-based approaches build a core ontology as a union of syntactic ontologies rather than as a semantically-rich description of the research domain in its own right [25-27]. In contrast, for rule-based mediation we use a core ontology, which is a biologically-relevant, tightly-scoped, logically-rigorous description of the semantics of the research domain. The core ontology describes the domain, in this case the specifics of the biology of interest. The core ontology may be created for this purpose or drawn from existing ontologies.

Because the core ontology is abstracted away from data formats, importing new data sources is made easier. Furthermore, the process of adding to the core ontology is simplified: each new mapping, class, or data import is incremental, without the need for large-scale changes. The richness of the core ontology depends on the type of biological questions that it has been created to answer; a detailed ontology may have higher coverage of the research domain, but may take longer to develop and reason over.

In contrast to global-as-view or local-as-view approaches, where either the target or source ontologies are entirely described using views of the other, the methods used in rule-based mediation provide a way to decouple syntactic and semantic heterogeneity and allow the core and syntactic ontologies to remain distinct. Additionally, rule-based mediation maps and loads data directly into the core ontology, allowing simplified querying via the use of standard tools, mapping and querying languages. Since the core ontology is more than just the entailment of a set of syntactic ontologies, it could be used for more than just rule-based mediation. Being a semantically-rich model of a research domain, it could also be used as a standalone ontology for marking up data or as a reference model of its biological domain. Furthermore, due to its independence from the syntactic ontologies, it is much more flexible with respect to changes.

#### Mapping between syntactic and core ontologies

The mapping in rule-based mediation is defined with the OWL-DL-based SWRL rule language. SWRL divides rules into a* antecedent* for describing the initial conditions of the rule, and a* consequent* for describing the target of the rule. Whenever the antecedent of the rule is true, the conditions in the consequent must also hold. When mapping from the syntactic ontologies to core ontologies, the antecedent of the rule is derived from a syntactic ontology, while the consequent corresponds to the mapped concept within the core ontology. Once data have been integrated into the core ontology, the knowledge contained within the core ontology can be reasoned over and queried, and the query response formatted according to a suitable syntactic ontology. The quality of the data exported back to a syntactic ontology, and in turn to a base format, is dependent upon both the quality of the mappings to the syntactic ontology and the syntactic ontology's scope. If either the mappings are incomplete, or the underlying data format has no way of describing the new data, the export will lack information.

SQWRL is a rule language based on SWRL, and is used for querying an OWL ontology and formatting the query response. In a way analogous with how SQL SELECT statements are used in a relational database management system, SQWRL presents the user with the results of a query, but does not store those results back in the ontology. Within the context of this paper, SQWRL queries are used to test the structure of a query or rule, and to illustrate interesting, but indirectly-relevant, queries. If results should be stored, SWRL mapping rules will save the results within the ontology.

### Rule-based mediation in the context of model annotation

Current systems biology model annotation tools have limited access to the semantics of the models. We have implemented rule-based mediation to address this limitation, creating a method of model annotation which imports information from data sources in a semantically- and syntactically-integrated way. This approach retrieves information that is tailored to the needs of the modeller and more relevant that that retrieved through syntactic methods. We have used a combination of existing tools, rule languages and novel mappings to perform proof-of-principle integration for two use cases.

**Figure 1:**
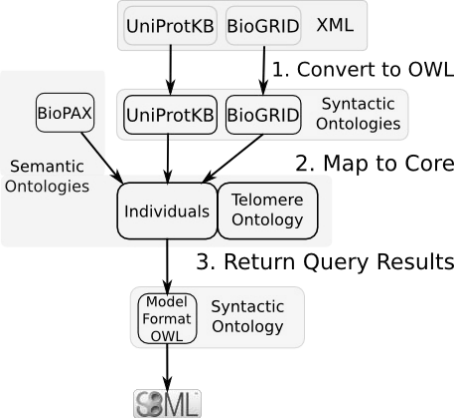
**Rule-based mediation in the context of SBML model annotation.** Non-OWL formats are first converted into syntactic ontologies. Here, both UniProtKB and BioGRID data are in XML: UniProtKB has its own schema, while BioGRID uses PSI-MIF. These formats are converted into syntactic ontologies via the XMLTab. BioPAX, the format used for Pathway Commons, is already in OWL and needs no conversion. Next, the instances present in the syntactic ontologies are mapped to the core ontology using SWRL. Finally, querying is performed using SQWRL queries using only core ontology concepts. Information is then passed through a final syntactic ontology (MFO) into an SBML model.

Figure 1 shows rule-based mediation in the context of SBML model annotation. First, information is syntactically converted into OWL-DL; second, the information is mapped into a core ontology; third, the core ontology is queried to answer specific biological questions; and finally, the new information is sent back to an external format, in this case SBML, via mappings from the core ontology to its syntactic ontology. From a biological perspective, rule-based mediation produces an integrated view of information useful for modelling.

In this paper we have extended a quantitative biological SBML model via rule-based mediation as a proof-of-principle for the methodology. To facilitate the use cases, we created a core ontology describing a subset of telomere biology. We then used three standard semantic web tools (XMLTab [28], SWRLTab [29] and SQWRLQueryTab [30]) to integrate heterogeneous data sources containing information relevant to this model. The information retrieved from the core ontology allowed us to annotate an SBML model, including the provisional identification of information not previously present in the model. This work demonstrates the feasibility of the approach, the applicability of the technology and its utility in gaining new knowledge.

#### Syntactic ontologies for the use cases

Rule-based mediation separates the resolution of syntactic and semantic heterogeneity into two steps. The first step is the syntactic conversion of a data format into a syntactic ontology. Four data sources were used to create four syntactic ontologies for this work: BioGRID [31] in PSI-MIF [32] format, Pathway Commons [33] in BioPAX format, BioModels [34] in SBML format and UniProtKB [35]. For each data format, a suitable syntactic ontology was either identified or created. BioModels was used to retrieve the SBML model to be annotated, while the other three were used as general data inputs to the integration system. However, BioModels can also be used as a general data input, which could result in the identification of links among models. Basic information on the data formats as well as the numbers of classes, relations and mapping rules in their syntactic ontologies is available in Table 1.

**Table 1:** Basic information about the syntactic ontologies and their mapping rules with the core ontology.

Data Source	Data Format	Classes	Object Properties	Data Properties	DL Expressivity	Rules
UniProtKB	UniProtKB XML	27	24	34	*ALEN* (D)	11
BioGRID	PSI-MIF	26	24	15	*ALEN* (D)	17
SBML	SBML	516	56	44	*SHOIN* (D)	14*
Pathway Commons	BioPAX	41	33	37	*ALCHN* (D)	11

Pathway Commons differs from the other sources as its format, BioPAX, is already represented in OWL. The ''Rules'' column lists the number of SWRL mapping statements used to link each syntactic ontology with the core ontology. For the SBML syntactic ontology, the numbers are combined totals of the imported Systems Biology Ontology and MFO. MFO was used for export only, and therefore its rules are marked with "*'' to show that these are export rules. Statistics generated using Protégé 4 (http://protege.stanford.edu/).

Previously we described Model Format OWL (MFO) as a syntactic ontology representing constraints from SBML, Systems Biology Ontology (SBO) and the SBML manual [36]. MFO has a dual purpose for the presented use cases: firstly, it was used as the format for the representation of any data stored as SBML and secondly, it was used to format query responses as SBML.

BioPAX was used in its native OWL-DL format. Information about the network neighbours of any given Pathway Commons entity can be accessed as a BioPAX document written in OWL-DL. Therefore, no additional syntactic ontology was needed since the populated BioPAX ontology returned from a nearest neighbour query could be used directly.

**Figure 2:**
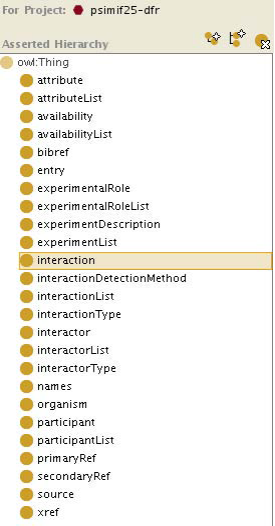
**Overview of the PSI-MIF syntactic ontology.** The PSI-MIF syntactic ontology is used for BioGRID data, and was created by the Protégé XMLTab plugin using the PSI-MIF-formatted result of a query over BioGRID.

**Figure 3:**
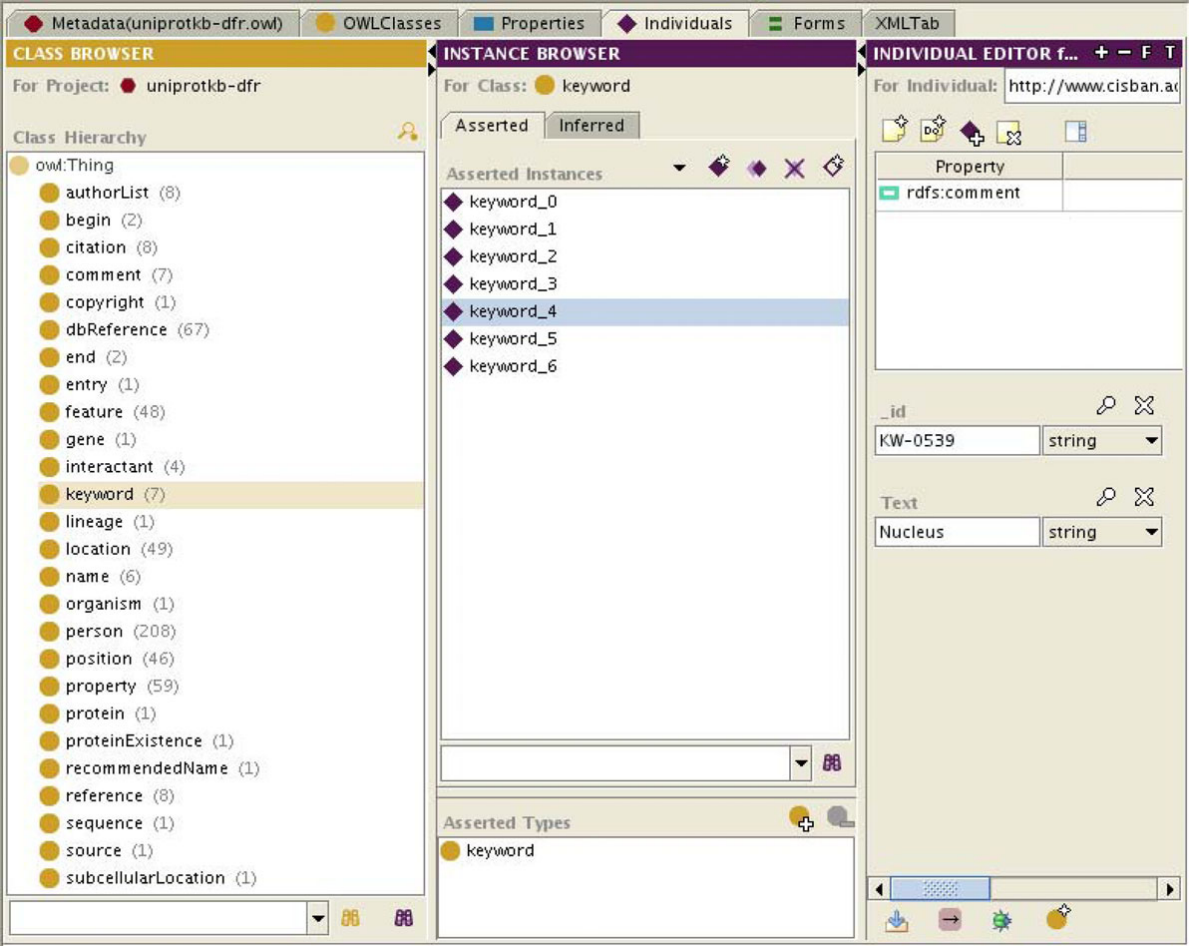
**The UniProtKB syntactic ontology.** The UniProtKB syntactic ontology represents the structure of UniProtKB entries. As with the other syntactic ontologies, instances represent the data itself while classes represent the structure.

The PSI-MIF and UniProtKB syntactic ontologies were created from their XML data formats. Ontology classes represent element and attribute types, while the data items themselves are represented with ontology instances. Figure 2 shows an overview of the classes created for the BioGRID syntactic ontology. As this work was being completed, Pathway Commons began importing BioGRID data. Therefore in future, Pathway Commons can be used to retrieve BioGRID data rather than the PSI-MIF syntactic ontology. However, the PSI-MIF syntactic ontology developed is not redundant: it can be used to integrate any other PSI-MIF-based data sources. Figure 3 shows an excerpt of the UniProtKB syntactic ontology. A UniProtKB syntactic ontology was generated using the UniProtKB XML format. Unambiguous assignment of a UniProtKB primary accession to a new species in an SBML document provided a useful way to annotate individual species as well as identify similar proteins. For the use cases, UniProtKB was primarily useful for localisation and identification information.

#### Telomere ontology

**Figure 4:**
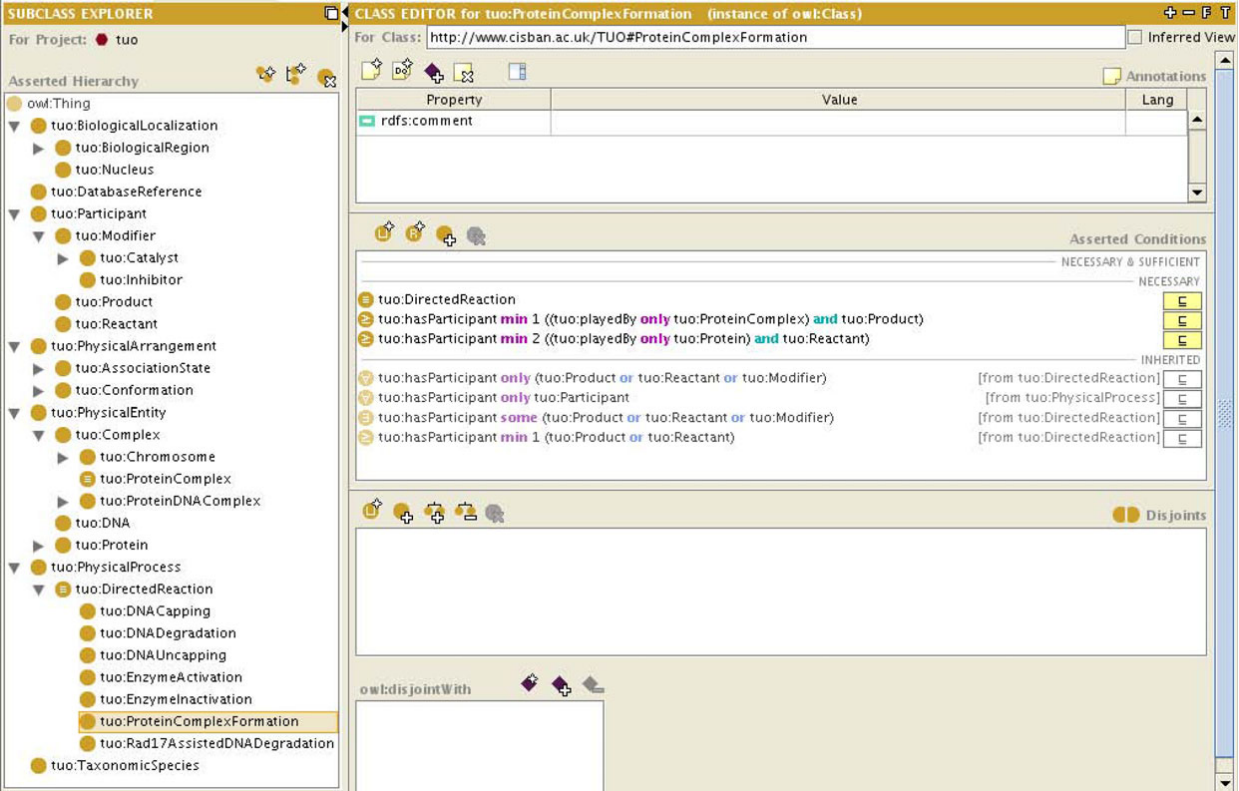
**An overview of the telomere ontology.** The telomere ontology is the core ontology for the use cases presented here.

Once the syntactic ontologies are created, the second step in rule-based mediation is the creation or identification of a suitable core ontology. Unlike the syntactic ontologies, which are designed to be OWL-DL representations of the underlying data formats, a core ontology is an explicit description of the semantics of the research domain. The core ontology created for these use cases is the* telomere ontology,* which models the biology relevant to the Proctor* et al.* model [37] of telomere uncapping. The telomere ontology was created to describe and aid the identification of pathways, genes and other biologically-relevant entities involved in ageing processes in yeast. Figure 4 shows a portion of this ontology. While a comprehensive telomere ontology is not yet complete, the parts necessary for the use cases have been fully constructed.

### Use cases

Rule-based mediation was performed for two use cases to show proof-of-principle for SBML model annotation. The mapping rules from the syntactic ontologies to the telomere ontology were run prior to performing the queries over the data. These rules populated the telomere ontology with the instance data from the syntactic ontologies. The telomere ontology, the syntactic ontology, the SWRL mappings, SQWRL queries, and the SBML model before and after annotation in these use cases are available on the project page at http://cisban-silico.cs.ncl.ac.uk/RBM/.

In this paper, we consider two methods for enriching an existing model of telomere uncapping in *Saccharomyces cerevisiae.* In Use Case 1, we annotate a single SBML species with information relevant to the gene* RAD9.* Adding information to existing SBML elements at an early stage aids model development and extension prior to publication or deposition. In Use Case 2, we retrieve possible protein-protein interactions involving the RAD9 protein. This approach resulted in the identification of model elements as well as a putative match for an enzyme that was not identified in the original curated model. These examples show how rule-based mediation works in a systems biology setting. The use cases also show that rule-based mediation successfully integrates information from multiple sources using existing tools, and that it would be useful to expand the implementation of this method to larger biological questions and automated model annotation.

#### Use Case 1: annotation

Use Case 1 demonstrates the addition of basic information to the species such as cross-references, SBO annotations, compartment localisations and a recommended name. For this use case we queried the telomere ontology for information about the* S.cerevisiae* query gene* RAD9.* Queries for proteins possessing 'rad9' as a name or synonym are stored within the telomere ontology as SWRL rules. An example rule is shown below. This rule is one of those used to collect all matching telomere ontology proteins and further declare them specifically as RAD9 proteins:

*tuo* : *Protein*(?*rules* : *someEntity*) Λ 

*tuo* : *synonym*(?*rules* : *someEntity,* ?*rules* : *s*) Λ 

*swrlb* : *containslgnoreCase*(?*rules* : *s*,* "rad9"*) → 

*tuo* : *Rad9*(?*rules* : *someEntity*)

The rule queries the synonyms of the protein names within the telomere ontology for a string matching “rad9”, and declares any matches as RAD9 proteins. In use, this and related rules uncovered three instances, each from a different data source: one from Pathway Commons, one from UniProtKB, and one from BioGRID. These three instances were then declared equivalent. The final step of this use case is to restrict the instances of RAD9 to the organism of interest,* S.cerevisiae.*

Once integrated, the information contained within these three instances is then sent out, using MFO, to a new version of the Proctor* et al.* SBML model. In order to export the relevant knowledge to MFO, 14 mapping rules with telomere ontology classes in the antecedent and MFO classes in the consequent were created. Four of the 14 were used specifically to create MIRIAM cross-references and SBO terms. The two Proctor* et al.* RAD9 species originally contained a single reference to UniProtKB, which was confirmed by the information retrieved from the telomere ontology. In addition, an SBO Term and five other MIRIAM annotations from Intact, UniProtKB, Pathway Commons and SGD were exported.

#### Use Case 2: interactions

Use Case 2 shows how possible protein-protein interactions can be retrieved to annotate models. Specifically, interactions involving RAD9 were retrieved from the telomere ontology, confirming those already present in the curated model as well as discovering novel interactions. The Proctor* et al.* model has four interactions between RAD9 and other proteins, as described in the Proctor* et al.* column of Table 2. Two of these interactions are with known proteins (RAD53 and CHK1), and two are with proteins whose identity was unknown to the modeller (labelled Rad9Kin and ExoX). Importantly, here, rule-based mediation has provisionally identified of one of those previously unidentified proteins. Execution of the strategy described resulted in the proposal of new model elements as well as the putative identity of an enzyme responsible for activation of RAD9. The results of Use Case 1 are used as part of the input in this use case. The SQWRL query shown below is for displaying, but not storing, every interaction involving RAD9.

**Table 2:** Summary of interactions retrieved for Use Case 2 against the core telomere ontology.

Discovered Interaction Partner with P14737	Proctor* et al.*	BioGRID	Pathway Commons
Serine/threonine-protein kinase RAD53 (P22216)	✓	✓	✓
Serine/threonine-protein kinase CHK1 (P38147)	✓		✓
Serine/threonine-protein kinase MEC1 (P38111)		✓	
DNA damage checkpoint control protein RAD17 (P48581)		✓	
Rad9Kin (*)	✓		
ExoX (*)	✓		

All discovered interactions already present in the curated model are shown, together with example interactions from the curated model that were not discovered (with Rad9Kin and ExoX) and discovered interactions that were* not* present in the model (with MEC1 and RAD17). SBML species shown with an asterisk (*) are those which are placeholder species, and therefore cannot have a match to a real protein. Some interactions are false positives inherent in the data source, while others are out of scope of the modelling domain of interest and should not be included.

*tuo* : *Rad9*(?*rules* : *rad9instance*) Λ

*tuo* : *plays*(?*rules* : *rad9instance*, ?*rules* : *participant*) Λ

*tuo* : *hasParticipant*(?*rules* : *process*, ?*rules* : *participant*) →

*sqwrl* : *select*(?*rules* : *rad9instance*, ?*rules* : *process*)

The above query identifies interactions involving RAD9 instances, and displays the name of the instance as well as the process in which it is involved. The use of SQWRL here rather than SWRL allows the more than 270 returned interactions to be viewed. These results are deliberately not saved in the asserted ontology as many are not meaningful for the model under study. Instead, the SQWRL query was used to determine the full set of results, and then SWRL rules were used to filter the interactions according to those species already present in the model. Protein instances were classified in a similar way to Use Case 1. The example SWRL query shown below classifies CHK1 protein instances:

*tuo* : *Protein*(?*rules* : *someEntity*) Λ

*tuo* : *synonym*(?*rules* : *someEntity*, ?*rules* : s) Λ

*swrlb* : *containsIgnoreCase*(?*rules* : *s, "chkl"*) →

*tuo : Chk1*(?*rules* : *someEntity*)

The combined results from the rule above together with 12 associated rules allowed four proteins of interest to be classified. Specific interactions of interest were then identified. The OWL axioms on the telomere ontology class for interactions between RAD9 and RAD53 are shown below as an example. This class is one of four defined classes which filter the RAD9 interactions.

*Rad9/Rad53 Interactions* :

*tuo* : *hasParticipant some* (*tuo* : *playedBy some tuo* : *Rad9*) Λ

*tuo* : *hasP articipant some* (*tuo* : *playedBy some tuo* : *Rad53*)

Table 2 shows a summary of the inference results from those four defined interaction classes.

There are a total of four interactions involving RAD9 in the curated model. Two of these interactions, with RAD53 and with CHK1, were confirmed. The RAD53 interaction was found in both BioGRID and in Pathway Commons, while the CHK1 interaction was only in Pathway Commons. The other two RAD9 interactions present in the model include placeholder species, which were created by the modeller to describe proteins whose identity was unknown. Because these proteins had no defining features, they could not be directly identified with the same methods used for RAD53 and CHK1. We provisionally identified one of those placeholder species, marked as 'Rad9Kin' in the model, as the protein MEC1 using data originating in BioGRID.

In the original curated model, the 'rad9Kin' species does not have a UniProtKB primary accession, as the model author did not know which protein activated RAD9. However, MEC1 is shown in the telomere ontology as interacting with RAD9 and is present elsewhere in the curated model reactions. Further, UniProtKB reports MEC1 as a kinase which phosphorylates RAD9. From this information, the model author now believes that MEC1 could be the correct protein to use as the activator of RAD9 [38]. RAD17 is another example of a protein present in the curated model, but not marked as an interacting partner of RAD9. However, BioGRID data shows an interaction between RAD9 and RAD17. This interaction may have been unknown to the modeller, and it may be that the curated model could be improved by the addition of a RAD9/RAD17 reaction.

Finally, the rules to export the new information from the telomere ontology to MFO, as described in Use Case 1, were executed. As a result, new species, reactions, and MIRIAM annotations were added to a new version of the original SBML model. Existing SBML species gained MIRIAM annotations, names and SBO terms. Two new reactions were also added: RAD9 with RAD17, and RAD9 with MEC1. The former was chosen to demonstrate how the formation of a protein complex is mapped back to MFO, and the latter was chosen as an example of the mapping of an interaction about which less is known. Furthermore, since MEC1 is a candidate for the value of the 'Rad9Kin' species in the curated model, a skeleton reaction in the annotated version of the curated model acts as a placeholder for manual curation. Further work by the modeller could then fill in the base reaction, species and annotation, including parameters, initial values and rate constants.

## Discussion

We have created a new method of semantic data integration called rule-based mediation that reduces the amount of manual effort required to annotate a systems biology model, and which allows access to the underlying semantics of the data. Rule-based mediation makes use of a semantically-rich core ontology together with mappings to and from syntactic ontologies that represent the data sources of interest. New data sources can be easily inserted without modifying the biologically-relevant core ontology. Separation of syntactic integration and semantic description of the biology is robust to changes to both the syntactic ontologies and the core ontology. The use of existing tools decreases development time and increases the applicability of this approach for future projects.

Rule-based mediation contains aspects of both global-as-view and local-as-view strategies. Global-as-view mapping defines a core ontology as a function of the syntactic ontologies rather than as a semantically-rich description of the research domain in its own right, though the level of dependence of the core ontology can vary [25-27]. However, defining a core ontology merely as a model of a set of data sources limits global-as-view's usefulness, as the ontology becomes brittle with respect to the addition of new data sources and new formats. With local-as-view, the core ontology is independent of the syntactic ontologies, and the syntactic ontologies themselves are described as views of the core ontology. However, query complexity increases quickly with local-as-view approaches, and data sources need to be forced into the structure of the global core ontology [20]. Both methods are limited in their ability to describe the nuances of the biological concepts being studied. Such information is often not captured, either because the syntactic ontologies are intended to model the data sources in global-as-view or, as in local-as-view, because the syntactic ontologies are just views over a core ontology, restricting the description of both the core biology and the syntax. Rule-based mediation solves these problems by separating the core and syntactic ontologies, benefiting from their independence.

### Syntactic heterogeneity

Syntactic heterogeneity was resolved by converting the four data formats into OWL-DL. When performing this step, issues of scalability, scope and provenance were considered carefully. For instance, XMLTab is not an ideal choice for long-term use, as it does not scale well, and cannot load multiple XML files at once into a single syntactic ontology. Additionally, it is not clear that the XMLTab project is in active development. However, tools such as the SWRLAPI's XMLMapper [39] or Krextor [40] may provide viable alternatives. Likewise, rules are currently manually-generated, which also scales poorly. This can be addressed by automatically generating many rules. While we have made extensive use of pre-existing applications, plugin, and libraries, we can foresee a time in the very near future where we will reach the limit of some of these technologies with respect to ontology size or the amount of imported data. Particularly, the large number of instances required in a larger-scale semantic data integration project will most likely necessitate the use of a database back-end for the ontologies. Some technologies are available for storing instances from ontologies within a database schema, such as Jena's database libraries [41], the Protégé database back-end [42], or that followed by Sahoo* et al.*[17].

Secondly, there are limitations in describing data provenance using the SBML standard syntax. When new MIRIAM annotations are added to an SBML model, there is no way of stating which procedure added the annotations, or the date they were added. At a minimum, the addition of the software type, version number and date on which the information was added should be included. The authors have been in discussions with the SBML community since the 2009 Hackathon to determine how best to solve this problem. Until this issue is resolved, no provenance information is included in the newly-annotated models.

The final issue to consider is scope: many data sources are able to export in multiple formats, but such formats may not provide the same set of information. Even though a particular format might be available, it may not contain the complete set of information present in the data source. For instance, BioModels data are available in many formats including BioPAX and SBML, and models from BioModels could be loaded into the telomere ontology via BioPAX. However, the native format for most BioModels is SBML, and conversion from this format is not lossless: for example, quantitative data such as rate laws are not supported in BioPAX. Therefore if BioModels database entries are to be annotated, input via MFO is best. Because the choice of data format can influence the type of data ultimately entering the core ontology, this syntactic phase of integration can also influence the semantic resolution step of rule-based mediation.

### Semantic heterogeneity

Semantic heterogeneity in rule-based mediation is resolved through precise mapping rules and a well-written core ontology. The biological domain of interest must be modelled appropriately, and the incoming data must be linked to appropriate concepts in the core ontology. However, semantic data integration is not simple, and there are a number of issues to be addressed when performing this step.

Firstly, in order to assert equivalences between instance data from the various data sources, the authors manually applied the owl:sameAs construct. This method is ill-suited to automated procedures. However, tool and reasoning support for OWL 2, now a W3C Proposed Recommendation [43], is growing. With OWL 2, some imminent advances such as the hasKey [44] construct will allow automation in this area.

Secondly, rules linking syntactic ontologies to the core ontology can be quite complex. Many rules in the work presented here have 14 or more clauses, but once a rule is in place, its execution is efficient and does not often need to be repeated. However, there are some limitations in the rule languages used. For instance, SWRL allows the creation of new ontology instances during rule execution. While this is useful, it can lead to unexpected results such as multiple identical individuals being created each time the rule is run. These limitations can be ameliorated through more sophisticated usages of OWL and SWRL tools. One such tool already used within the MFO project is the OWLAPI [45], which allows finer control over ontologies and their mapping and reasoning. Greater use of the SWRLAPI [39] might also address these limitations.

A type of semantic heterogeneity that can be difficult for any integration system to address is the use of two distinct data items from the one data source to describe a single concept from another, e.g. where a protein is described by two UniProtKB entries but only a single SBML species. In rule-based mediation, we represent this heterogeneity rather than reconcile it. However, such cases have not been created by this work, only present via imports from existing sources. The opposite case, where a single data item is used to annotate multiple concepts, is common in SBML, for example with active or inactive species. This is specifically allowed by the telomere ontology.

Finally, an understanding of the semantics of the data formats is required when developing the export mapping rules from the core ontology to a syntactic ontology. A simple case would be how different formats define similar concepts. For instance, a protein in BioPAX is strictly defined as having only one polypeptide chain, while a protein in UniProtKB can consist of multiple chains. A more complex case is when two concepts, e.g. two interaction instances, have virtually indistinguishable properties, making it difficult for automated procedures to know which interaction to use. Another challenge is addressing cases where concepts in the core ontology could map to more than one concept in the syntactic ontology. For example, in most systems biology formalisms there is a clear delineation between a protein as an abstract concept and a protein as a specific interactor in a reaction. Within SBML, this is defined using the speciesType or species elements for the concept of a protein, and speciesReference for the interactor. Within BioPAX level 2, this is exemplified by physicalEntity and physicalEntityParticipant. A similar dichotomy exists within the telomere ontology. When converting the information retrieved in Use Case 1 back to SBML, these considerations need to be taken into account. Ultimately, the use of speciesType is not ideal, as future versions of SBML will have a different way of modelling such concepts [46]. Therefore, to align with future versions of SBML as well as to make the mapping simple, the best solution is to link to the appropriate attributes of an SBML species element.

## Conclusions

Manual SBML model creation and annotation can be a slow process, and is made more difficult by the large number of data sources relevant to modelling. We have shown that rule-based mediation is capable of resolving both syntactic and semantic heterogeneity. Specifically, addressing syntax and semantics separately allows the straightforward addition of new information; the modelling of the biological domain of interest without any knowledge of underlying formats; and simple manipulation of the mapping rules using off-the-shelf tools.

We showed a proof-of-principle application of rule-based mediation to systems biology model annotation, producing a new SBML model containing new MIRIAM annotations, new species, and new reactions. Syntactic ontologies for the UniProtKB and the PSI-MIF formats were created, while MFO was updated and BioPAX was used without modification. Additionally, a telomere ontology was developed to model the biology associated with the use cases. In the course of this work, not only have new biological annotations been added, but we have discovered new biology by putatively identifying a previously-unknown protein in the Proctor* et al.* model. Future work will use this approach as the core of an automated semantically-aware model annotation system.

## Authors' contributions

ALL developed rule-based mediation and the described ontologies, and drafted the manuscript. PL, MP and AW participated in the development of the integration technique and ontologies. PL and AW helped to draft the manuscript. All authors read and approved the final manuscript.

## Competing interests

The authors declare that they have no competing interests.
